# Supplementary material for: Single-cell atlas of rainbow trout peripheral blood leukocytes and profiling of their early response to infectious pancreatic necrosis virus
Source: Front Immunol. 2024 Jul 5;15:1404209. doi: 10.3389/fimmu.2024.1404209 (PMC11258392; doi:10.3389/fimmu.2024.1404209)
Supplement: Supplementary file 7 [file Presentation_1.ppt]

## Slide 1
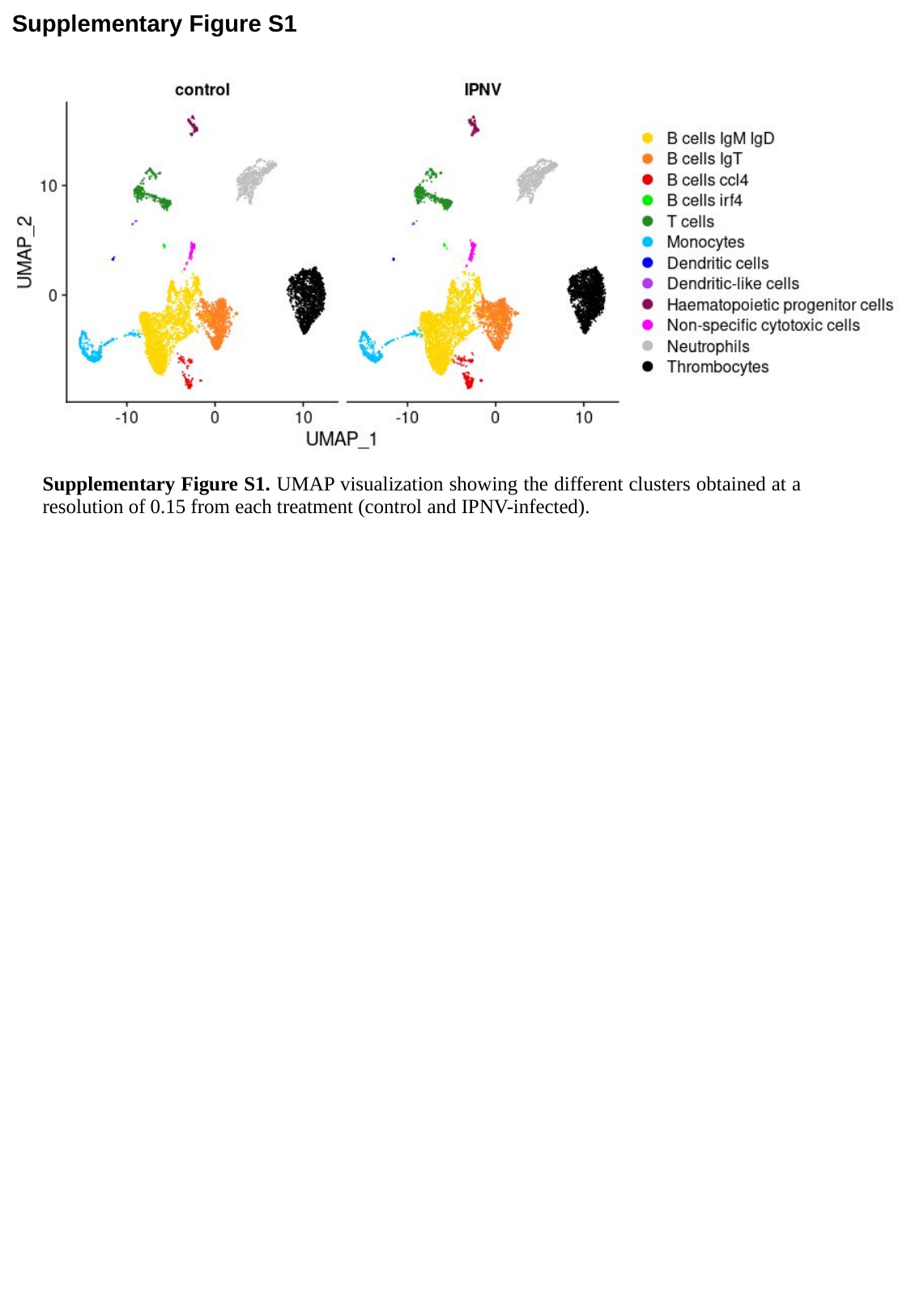

Supplementary Figure S1
Supplementary Figure S1. UMAP visualization showing the different clusters obtained at a resolution of 0.15 from each treatment (control and IPNV-infected).

## Slide 2
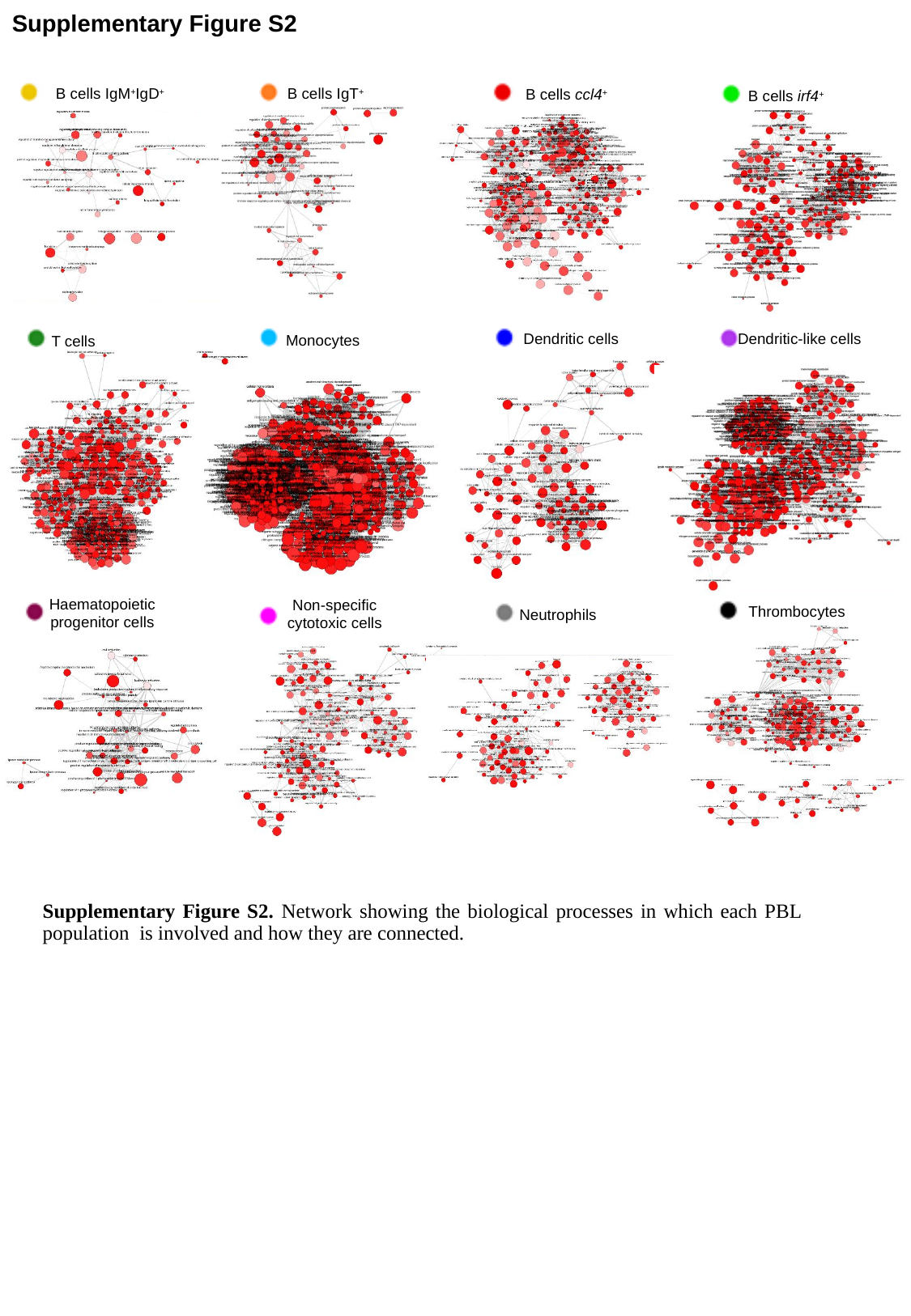

Supplementary Figure S2
B cells IgM+IgD+
B cells IgT+
B cells ccl4+
B cells irf4+
Dendritic cells
Dendritic-like cells
Monocytes
T cells
Haematopoietic progenitor cells
Non-specific cytotoxic cells
Thrombocytes
Neutrophils
Supplementary Figure S2. Network showing the biological processes in which each PBL population is involved and how they are connected.

## Slide 3
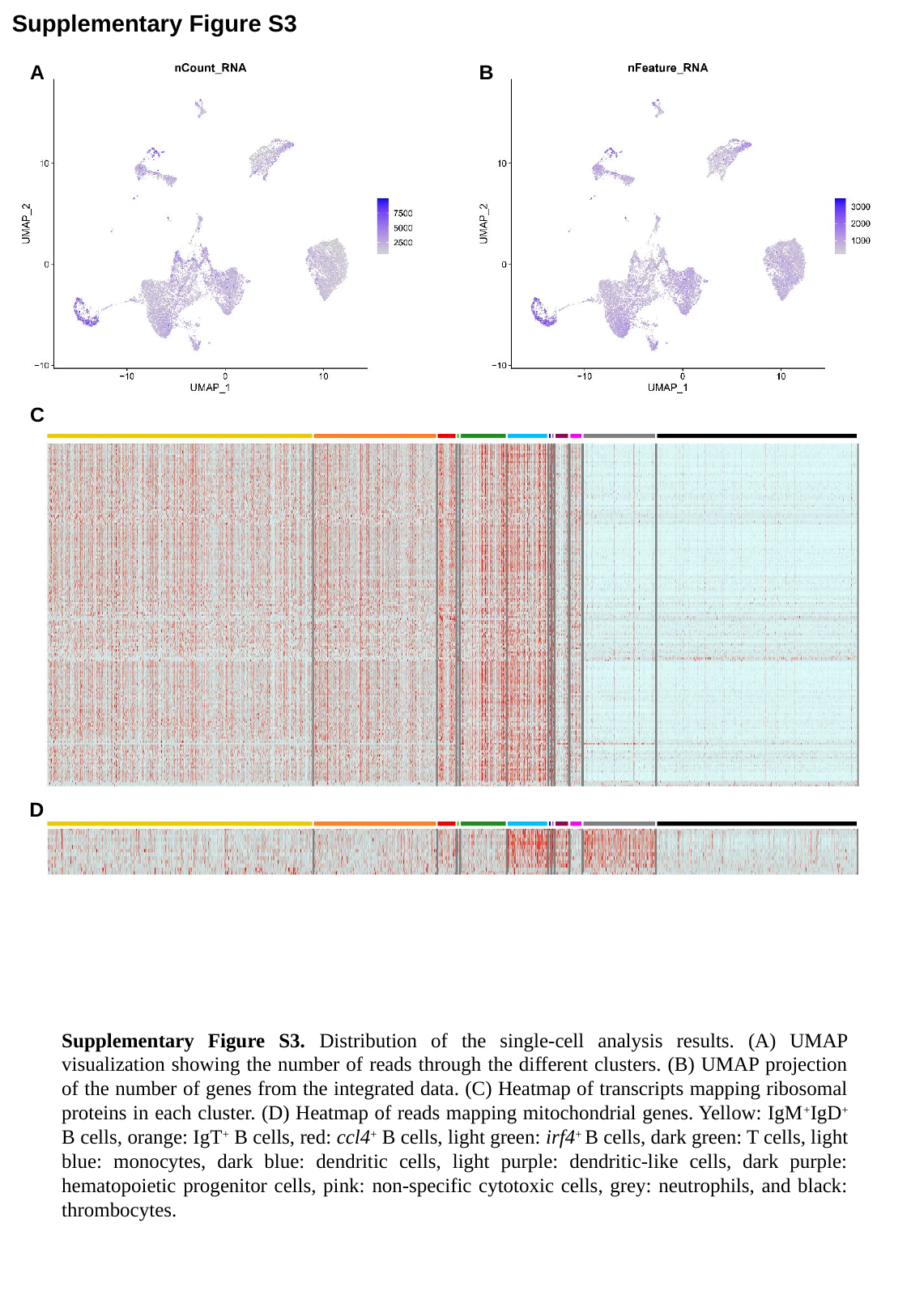

Supplementary Figure S3
A
B
C
D
Supplementary Figure S3. Distribution of the single-cell analysis results. (A) UMAP visualization showing the number of reads through the different clusters. (B) UMAP projection of the number of genes from the integrated data. (C) Heatmap of transcripts mapping ribosomal proteins in each cluster. (D) Heatmap of reads mapping mitochondrial genes. Yellow: IgM+IgD+ B cells, orange: IgT+ B cells, red: ccl4+ B cells, light green: irf4+ B cells, dark green: T cells, light blue: monocytes, dark blue: dendritic cells, light purple: dendritic-like cells, dark purple: hematopoietic progenitor cells, pink: non-specific cytotoxic cells, grey: neutrophils, and black: thrombocytes.
